# Interface Hardness Analysis of between IN625 and CoCrMo Manufactured by Pulsed Wave Laser Powder Bed Fusion

**DOI:** 10.3390/mi15010162

**Published:** 2024-01-21

**Authors:** Zhiong Sheng Hoo, Zhongmin Xiao, Liming Yao, Bozhong Jing, Chuanjie Jin, Chao Tang

**Affiliations:** 1 School of Mechanical and Aerospace Engineering, Nanyang Technological University, 50 Nanyang Avenue, Singapore 639798, Singapore; zhiongsh001@e.ntu.edu.sg (Z.S.H.); jinb0001@e.ntu.edu.sg (B.J.); 2 State Key Laboratory of Robotics and Systems, Harbin Institute of Technology, Harbin 150080, China; jinchuanjie666@163.com; 3 Zhengzhou Research Institute, Harbin Institute of Technology, Zhengzhou 450000, China; 4 Institute of High Performance Computing (IHPC), Agency for Science Technology and Research (A*STAR), Singapore 138632, Singapore

**Keywords:** additive manufacturing, L-PBF, dissimilar alloy hardness, pulsed wave laser, petrochemical

## Abstract

The nuclear and petrochemical industries often require multi-metal parts that are corrosion-resistant, heat-resistant, and possess high strength to enhance equipment safety and reduce downtime. Additive manufacturing technology enables the rapid and flexible processing of multi-metal parts to meet these stringent demands. This study is aimed at investigating the interface hardness between CoCrMo/IN625 to determine optimal processing parameters that can be utilized in manufacturing reliable and durable multi-metal parts. The result indicates that when the volumetric energy density, *E*_v_, is at or below 20 J/mm^3^, microfluidic forces are unable to sufficiently diffuse between the two metals, leading to insufficient diffusion, and the high hardness CoCrMo acts as a support, resulting in a significantly higher interface hardness. As *E*_v_ increases, intense recoil pressure within the microfluidic forces disrupts the melt pool, allowing for full diffusion between the two metals. The fully diffused high-hardness CoCrMo has been diluted by the low-hardness IN625, thus reducing the interface hardness. Considering the interface hardness, strength, and printing efficiency (time and energy consumption), we recommend a range of 35 J/mm^3^ < *E*_v_ ≤ 75 J/mm^3^. In this range, the average values for interface hardness and tensile strength of the samples are approximately 382 HV and 903 MPa, respectively.

## 1. Introduction

**Figure 1:**
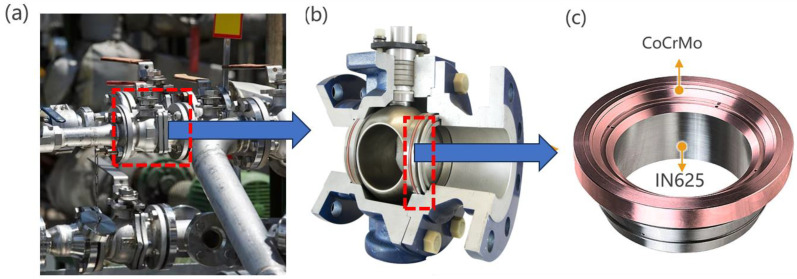
(**a**) Pipeline systems in the chemical industry; (**b**) ball valve; (**c**) valve seat ring.

In the aerospace, petrochemical, and nuclear industries, the majority of the components operate in harsh environments, and are extremely vulnerable to wear and corrosion, as well as thermal fatigue damage. For example, the acidic liquid flowing in the valves of a chemical plant can easily corrode the valve core, thus requiring special corrosion resistance treatment (Figure 1). Often, multiple components need to be assembled together or additional spraying and strengthening treatment [1,2,3,4]. Conventional techniques aimed at bolstering corrosion resistance, including fusion welding and the application of hot or cold coatings, are often ineffective for long-serving valve components and similar parts when it comes to utilizing dissimilar alloy joining techniques [5,6,7,8]. This is because the corrosion-resistant alloy coating is very thin and not resistant to erosion, and the interface area between the two alloys is weak, which is likely to be the site of cracks due to local strain and cannot be used for a long time. Co-based alloys exhibit high temperature resistance, excellent wear resistance, and good wettability with nickel-based alloys during the laser cladding process, making them a viable and robust option for securely amalgamating these two metals [9,10,11,12]. As an alternative to these, in order to extend the service life of current valves, improve the metal’s erosion corrosion resistance, reduce plant downtime, and reduce operating costs [13,14,15], the IN625 valve core base is covered with 10 mm-thick CoCrMo through laser-based powder bed fusion (LPBF) technology.

The LPBF process is a common method in metal additive manufacturing (AM), using high-energy laser beams to sequentially melt layers of powder and create three-dimensional (3D) components with intricate geometry [16,17]. For corrosion-resistant multi-metal LPBF, the focus is on integrating metals with high corrosion resistance, such as stainless steel, nickel-based alloys, or titanium, into the component’s design. Diverse combinations of metals can be investigated to generate cooperative outcomes that amplify resistance against corrosion. For instance, stainless steel and aluminium alloy bimetallic materials offer high corrosion resistance and lightweight properties for aerospace and medical components [18]. In biomedical industries, NiTi-Ti6Al4V multi-material implants provide improved corrosion and wear resistance [19]. Cobalt–chromium alloys combined with tungsten carbide or other hard particles offer excellent resistance to abrasive wear and impact. Nickel-based superalloys can be integrated with ceramics or diamond particles to create erosion-resistant surfaces in extreme environments [20,21]. Design plays a critical role in maximizing corrosion resistance. The LPBF process allows engineers to design complex geometries with internal channels and lattice structures that facilitate fluid flow and reduce stagnant zones where corrosion can initiate [22,23]. Moreover, functionally graded materials (FGMs) can be designed, where the composition gradually changes from one metal to another, resulting in a smooth transition of properties and enhanced corrosion resistance. Tan et al. initially employed selective laser melting (SLM) to fabricate a W-Cu (tungsten–copper) FGM. They discussed the influence of laser parameters on microstructure, interfacial defects, and bonding strength. The investigation revealed that irregular-shaped pores and cracks were the primary defects at the interface, attributed to the intrinsic properties of the materials [24]. In the investigation of SLM-fabricated SS316L conducted by Tucho et al., the porosity was found to be an inverse function of hardness. Within an energy density range of 50 to 80 J/mm^3^, the porosity exhibited a nearly exponential decrease, while hardness increased linearly with rising energy density. This phenomenon was primarily attributed to the collapse of pores within the material under load [25,26]. Analysing the interface hardness aids in enhancing the overall performance and reliability of these components, ensuring they can withstand extreme conditions without compromising safety or efficiency. Understanding the interface hardness between these materials is crucial, especially in additive manufacturing where multi-metal parts are increasingly used. Studying the interface hardness helps in evaluating the compatibility and performance of such combinations, which is vital for designing complex components in various industries [27,28,29]. Demir et al. presented their work on a multi-material SLM platform, demonstrating its application in producing Fe/Al-12Si multi-material structures. The layers of Fe/Al-12Si exhibited significant cracks due to the low compatibility and miscibility of these two materials. However, the obtained Fe/Al-12Si layers showcased high hardness, characteristic of the FeAl intermetallic [30]. By leveraging the capabilities of LPBF to combine multiple materials, engineers can create components with tailored erosion-resistant properties, leading to improved durability and reduced maintenance costs in demanding applications [4,31,32]. 

Currently, the predominant heating method in prevalent LPBF systems involves the use of continuous wave (CW) lasers. However, a few industrial systems opt for pulsed wave (PW) lasers as the primary heat source [33]. It is worth noting that the high power density produced by the PW laser, together with the vaporization and recoil effects, help to induce a good metallurgical bond between the two metals in a small heat-affected zone [34,35,36]. However, the hardness between IN625 and CoCrMo have not been studied using commercial PW lasers in the existing literature [37]. Therefore, we used a PW laser to deposit 10 mm-thick CoCrMo on the IN625 substrate to study the effect of different volumetric laser density, *E*_v_, on the interface hardness between IN625 and CoCrMo. Additionally, we utilized tensile strength data obtained from Yao et al.’s research [38] to support our investigation. We aimed to recommend optimal *E*_v_ values that ensure high reliability and durability of multi-metal components by achieving a desired interface hardness while preserving high strength.

## 2. Materials and Methods

### 2.1. Materials

A 200 mm × 200 mm × 25 mm IN625 substrate and spherical CoCrMo powder, ranging in particle size from 10 µm to 45 µm, were employed in this investigation. These materials’ chemical compositions are demonstrated in Table 1.

### 2.2. Sample Printing Process

**Figure 2:**
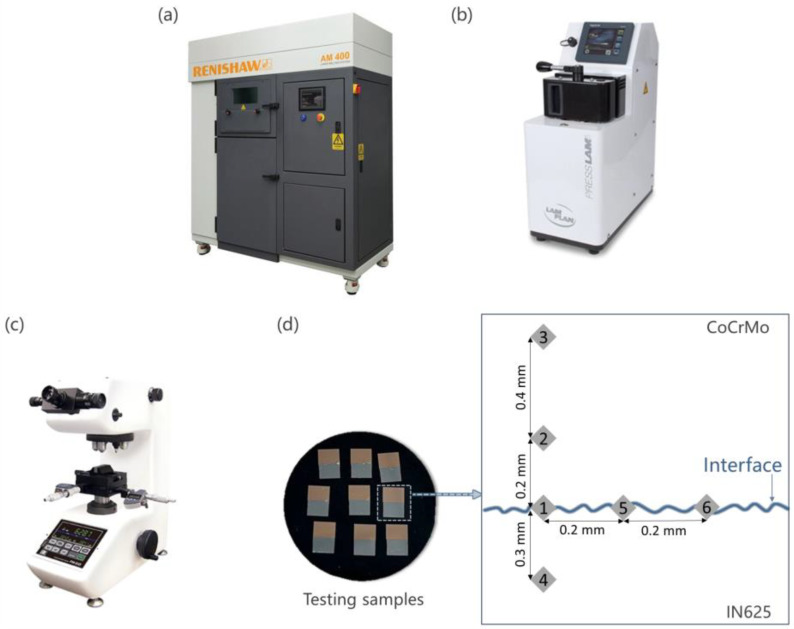
(**a**) Renishaw AM400 LPBF printer, (**b**) PRESSLAM 1.1 hot mounting press, (**c**) FM300e hardness tester, and (**d**) testing samples with indicated positions of Points 1, 2, 3, 4, 5, and 6 where hardness being measured are shown alongside. Silvery grey CoCrMo is visually represented in brown colour in this figure to aid distinction.

**Figure 3:**
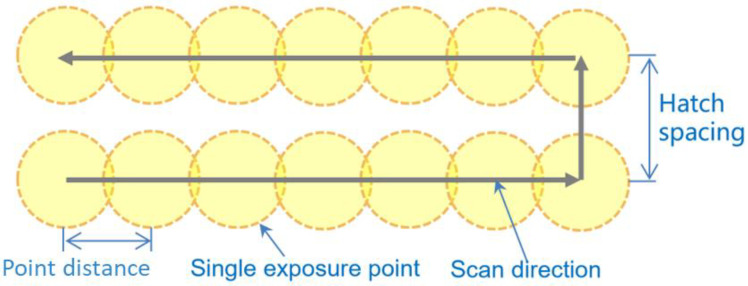
Fundamental operation and essential factors of a PW laser.

The metal 3D printer (AM400, Renishaw plc, Kingswood, UK) shown in Figure 2a was utilized to manufacture CoCrMo blocks on a square IN625 substrate. Operating in a power-modulated PW mode, a fibre laser with a 1070 nm wavelength and a 70 μm laser diameter was utilized by the AM400. As shown in Figure 3, this mode allowed precise control over hatch spacing, point distance, laser power, and exposure time. The experiment comprised 27 distinct pulse parameter combinations outlined in Table 2. The velocity of laser scanning *V*, derived from the division of point distance, $P_{d}$, by the time of exposure, *E*_t_, ranged from 0.36 m/s to 2.00 m/s. Post printing of each powder layer, the laser scan direction was altered by 90° without implementing any boundary scan. The parameters of 60 µm for powder layer thickness, *T*, and 80 µm for hatch distance, $H_{s}$, were included. A 200 mm × 200 mm × 25 mm IN625 substrate with a sand-blasted surface was secured on the building platform inside the machine chamber, followed by heating to 90 °C. A recoater with a rubber blade was used to deposit layers of powder on the substrate. Protective inert argon gas was introduced into the chamber to reduce the interior oxygen level to 0.1%. Approximately 5 mm-thick CoCrMo was printed on the substrate, which was then wire-cut to create 54 small testing samples, each with dimensions of 10 mm × 6 mm × 6 mm as highlighted in the dotted box in Figure 2d.

Unlike CW lasers, the input of PW laser operates intermittently, impacting the energy input. The measurement of volumetric energy density (*E*_v_) is crucial in evaluating laser energy input, it represents the average applied energy per unit volume of metals during the scanning of one layer [39]. Equation (1) addresses the calculation for volumetric energy density, *E*_v_ (J/mm^3^), with a CW laser emitter [40]. However, adjustments are necessary for pulsed laser emitters, as shown in Equation (2), ensuring an accurate quantification of the parameter combinations detailed in Table 2.

(1) $$E_{V}=P/\left(V\times H_{s}\times T\right)$$

(2) $$E_{V}=\delta \times P/\left(V\times H_{s}\times T\right)$$

In this context, laser power is denoted by *P* (W), hatch spacing by $H_{s}$ (μm), scan velocity by *V* (m/s), point distance by $P_{d}$ (μm), and exposure time by $E_{t}$ (μs). In Equation (2), δ represents the duty cycle, which spans from 0.0 to 1.0. Its role involves amplifying the pulsed wave (PW) exposure factor. As per Brown et al.’s research, duty cycles at 0.54, 0.75, and 0.90 align with exposure durations of 50 μs, 80 μs, and 110 μs, respectively [15]. Table 2 shows the resulting *E*_v_ calculated using Equation (2). Upon depositing CoCrMo onto the IN625 substrate, variation in composition and thermal characteristic between the materials might lead to weakened areas near the interface. To explore this concept, the process parameters detailed in Table 2 were employed using two distinct approaches. The first approach encompassed the printing of successive layers of powder without the process of double laser melting within the same layer, while in another approach, only the first three layers underwent double melting.

### 2.3. Microhardness Test

The hot mounting press (PRESSLAM 1.1, Lam Plan S.A.S., Gaillard, France) was employed for the production of moulds used in microhardness testing. The process of hot mounting involves creating moulds that securely hold the samples in place during subsequent polishing procedures. Before mounting, the printed parts were cleaned with alcohol to improve adhesion to the mounting resin. Subsequently, 9 dried printed parts were subjected to hot mounting with Phenolic 642 resin in a single application, as illustrated in Figure 2b. A heating time of 3.5 min and a heating temperature of 170 °C were set to induce a phase change in the resin, facilitating the embedding of printed parts with an applied pressure of 800 daN. A subsequent cooling period of 3 min solidified the resin, forming a stable mould. A total of 6 resin cylinders were yielded, each around 25 mm in diameter. The LaboForce-50 (Streurs S.A.S., Champigny sur Marne, France) was used to perform polishing of the samples. The polishing wheel was set to a speed of 300 RPM. The SiC papers used for polishing were 220-, 500-, 1200-, 2000-, and 4000-grit papers. Then, finely grind using MD-Largo (DiaPro Largo 9 μm suspension, Streurs S.A.S., Champigny sur Marne, France). The scratch-free finish was obtained, and the sample was taken for microhardness testing.

The Vickers Microhardness Test was conducted using the hardness tester (FM300e, Future-Tech Corp., Kawasaki, Japan). Then, we set the load to 300 gf. This was kept constant during the entire experimental process for all the readings of all 54 samples. The average indentation dwell time was 10 s. Six measurements were taken at various points along the specimen’s cross-section for the microhardness analysis. Points 1, 5, and 6 were taken along the interface with a separation distance of 0.2 mm. Two hardness readings were taken on the sample’s powder (CoCrMo) end. Points 2 and 3 were taken at a distance of 0.2 mm and 0.6 mm measured vertically from Point 1. Another reading was taken at a distance of 0.3 mm (Point 4) measured vertically from Point 1 towards the substrate (IN625) side. This was repeated for all 54 samples, as shown in Figure 2d. The average readings of Points 1, 5, and 6 portrayed the interface hardness. Both Points 2 and 3 represented the hardness of the printed CoCrMo, whereas Point 4 showed the hardness of the IN625 substrate.

## 3. Results and Discussion

**Figure 4:**
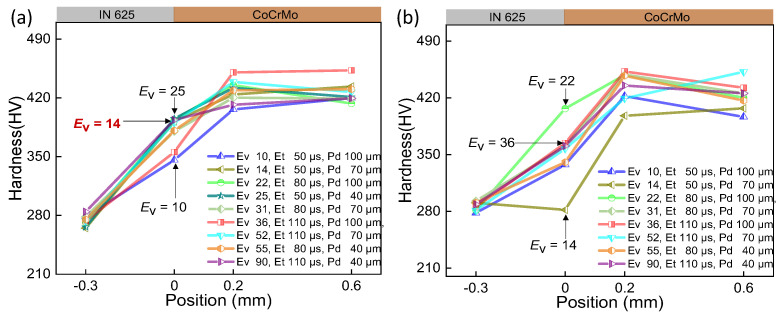
The interface hardness of CoCrMo/IN625 within the transition range at the laser power of 175 W. (**a**) Layer-by-layer printing of powder without double melting; (**b**) only the first three layers of powder experience double laser melting.

**Figure 5:**
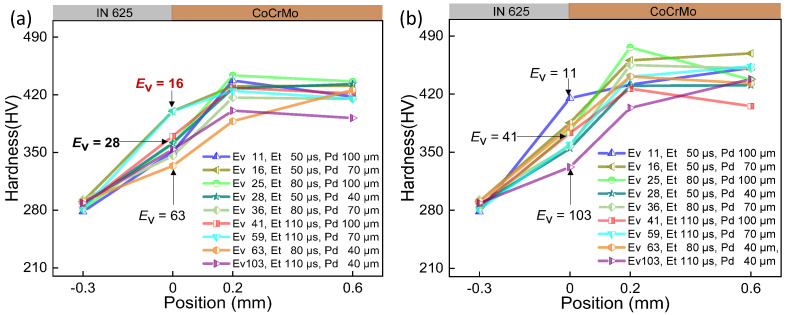
The interface hardness of CoCrMo/IN625 within the transition range at the laser power of 200 W. (**a**) Layer-by-layer printing of powder without double melting; (**b**) only the first three layers of powder experience double laser melting.

**Figure 6:**
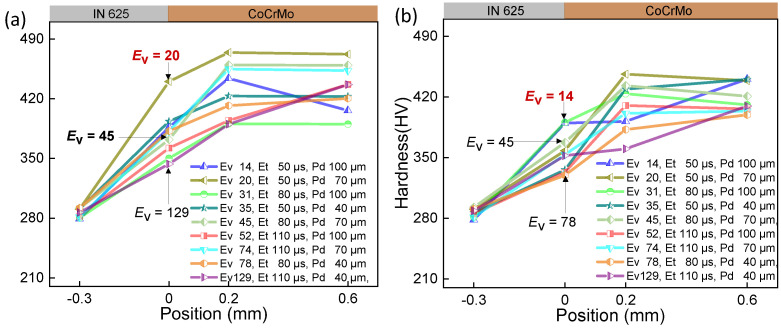
The interface hardness of CoCrMo/IN625 within the transition range at the laser power of 250 W. (**a**) Layer-by-layer printing of powder without double melting; (**b**) only the first three layers of powder experience double laser melting.

**Figure 7:**
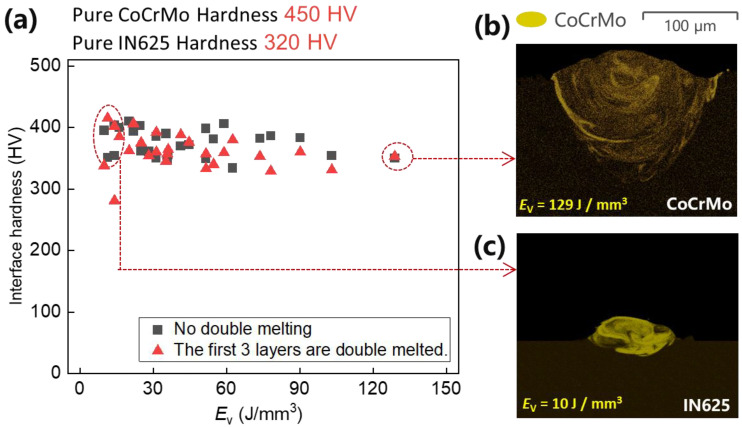
The changes in hardness (**a**) observed in the CoCrMo/IN625 sample, influenced by the *E*_v_. (**b**,**c**) depict metal component distribution captured using Energy-Dispersive X-ray Spectroscopy (EDS) in cross-sections perpendicular to the build and scan direction.

Extensive experimental work is required to determine the processing windows for novel material systems, considering the distinct laser absorbance and thermal properties inherent to each material. These processing parameters play a crucial role in obtaining the intended properties for the production of parts. The curves of hardness for samples under various parameters were displayed in Figure 4, Figure 5 and Figure 6, while the impact of volumetric energy density on hardness is illustrated in Figure 7.

In Figure 4, Figure 5 and Figure 6, it is evident that Point 4 (−0.3 mm), where the hardness test was conducted on the IN625 substrate site, consistently shows IN625 hardness ranging from 270 HV to 300 HV across all 54 samples. This uniformity suggests that the test point remains unaffected by and is situated outside the heat-affected zone or fusion zone resulting from the laser printing of CoCrMo on the substrate surface. On the printed CoCrMo site (Points 2 and 3), the measured hardness varies significantly, ranging from 360 HV to 473 HV. This substantial hardness variation can be attributed to the different processing parameters, as detailed in Table 2. The energy and subsequent temperature the laser have imparted to the material cause variations in heating/cooling rates. It is clear that by optimising the volumetric laser energy density input, the as-manufactured hardness of the printed material can be controlled [26].

The comparison between (a) and (b) in Figure 4, Figure 5 and Figure 6 reveals a similar trend in interface hardness variations between IN625 and CoCrMo concerning changes in *E*_v_. When *E*_v_ is at or below 20 J/mm^3^, considered inadequate, it results in a small melt pool with a depth of 25 μm and a width of 102 μm, as depicted in Figure 7c. Microfluidic forces (Marangoni forces and surface tension) struggle to facilitate adequate transfer between the two metals, hindering sufficient diffusion between them. Upon pressing the indenter of the hardness tester against the middle of the interface fusion zone, the insufficiently diffused high-hardness CoCrMo provides support, leading to a notably higher interface hardness. However, due to the presence of low-hardness IN625 near the interface, the interface hardness is lower than pure CoCrMo hardness (450 HV), ranging between 350 and 420 HV, with the maximum hardness close to the CoCrMo hardness.

As *E*_v_ > 20 J/mm^3^, hardness gradually decreases with the rising laser volumetric energy density. This occurs because an increase in *E*_v_ results in larger melt pool depth (152 μm) and width (210 μm), allowing microfluidic forces (Marangoni forces and surface tension) to exert greater influence. In particular, the intense recoil pressure generated after metal vaporization disrupts the melt pool significantly, promoting thorough diffusion between the metals (Figure 7b). Upon pressing the indenter of the hardness tester against the middle of the interface fusion zone, the fully diffused and dissolved high-hardness CoCrMo gets diluted by the low-hardness IN625, leading to a noticeable reduction in interface hardness. As the laser volumetric energy density (*E*_v_ = 129 J/mm^3^) increases, the dilution of high-hardness CoCrMo becomes more pronounced, resulting in a smaller interface hardness. At this point, the interface hardness ranges between 331 HV and 390 HV, with the minimum hardness close to the IN625 hardness (320 HV). To enhance understanding of *E*_v_’s influence on the interface hardness of the two metals, Figure 7a was generated using data from Figure 4, Figure 5 and Figure 6. As anticipated, increased *E*_v_ corresponds to heightened alloy amalgamation (Figure 7b,c), resulting in a gradual reduction in interface hardness.

However, elevated interface hardness does not necessarily signify superior sample quality. A comprehensive evaluation of sample quality requires considering sample tensile strength. Yao et al.’s tensile strength test results for IN625/CoCrMo were incorporated [38]. It becomes evident that at *E*_v_ ≤ 20 J/mm^3^, the interface strength is notably low, with fractures occurring on the CoCrMo side. At *E*_v_ = 10 J/mm^3^, CoCrMo exhibits voids exceeding 200 μm in width, leading to reduced CoCrMo strength, generally below 230 MPa, and inferior interface quality due to 10 μm-wide voids. However, the presence of IN625 near the interface reduces the difficulty of CoCrMo melting, resulting in smaller void sizes on the interface compared to those within CoCrMo. Lower *E*_v_ conditions also facilitate melting of IN625/CoCrMo, leading to interface strength surpassing that of the CoCrMo component, thus causing fractures in the CoCrMo portion. In the range 10 J/mm^3^ < *E*_v_ ≤ 20 J/mm^3^, fractures emerge near the interface, where the interface strength escalates with *E*_v_, while the hardness diminishes. Beyond *E*_v_ > 30 J/mm^3^, the interface strength remains unaffected, while the hardness gradually decreases with an increasing *E*_v_. A marginal reduction in the interface hardness is observed for the first three layers of powder after remelting, as depicted in Figure 7a. This reduction is attributed to enhanced diffusion between CoCrMo/IN625 following double melting process of the first three layers, contributing to reduced interface hardness.

Considering interface hardness, strength, and printing efficiency (time and energy), a suggested range is 35 J/mm^3^ < *E*_v_ ≤ 75 J/mm^3^, where the average tensile strength reaches approximately 903 MPa, and the average interface hardness stabilizes around 382 HV.

## 4. Conclusions

In this study, using LPBF, we investigated the impact of different printing parameters, specifically laser volumetric energy density, *E*_v_ on the interface hardness between IN625 and CoCrMo. We explored 27 printing parameters and two melting approaches, analysing the relationship between *E*_v_ and hardness. For *E*_v_ values ≤ 20 J/mm^3^, we observed a shallow melt pool depth (25 μm) and narrow width (102 μm), hindering sufficient diffusion between metals. This led to an increased interface hardness due to unsupported high-hardness CoCrMo, but lower than pure CoCrMo hardness (450 HV). *E*_v_ increments increased the melt pool depth (up to 152 μm) and width (up to 210 μm), enabling thorough diffusion. However, highly diffused CoCrMo diluted by low-hardness IN625 resulted in reduced interface hardness.

Considering interface hardness, strength, and printing efficiency, we recommend a range of 35 J/mm^3^ < *E*_v_ ≤ 75 J/mm^3^. Within this range, samples exhibited an average tensile strength of approximately 903 MPa and an average interface hardness of around 382 HV.

## Figures and Tables

**Table 1:** (**a**) Chemical compositions of IN625 substrate. (**b**) Chemical compositions of CoCrMo powder.

(**a**)		
**Element**	**Chemical Formula**	**Chemical Composition (%)**
Nickel	Ni	Balance
Chromium	Cr	20.00–23.00
Molybdenum	Mo	8.00–10.00
Niobium	Nb	3.15–4.15
Titanium	Ti	≤0.40
Aluminium	Al	≤0.40
Iron	Fe	≤5.00
Copper	Cu	≤0.07
Cobalt	Co	≤1.00
Carbon	C	≤0.10
Manganese	Mn	≤0.50
Sulphur	S	≤0.015
Phosphorus	P	≤0.015
Silicon	Si	≤0.50
(**b**)		
**Element**	**Chemical Formula**	**Chemical Composition (%)**
Cobalt	Co	Balance
Chromium	Cr	27.00–30.00
Molybdenum	Mo	5.00–7.00
Silicon	Si	0.60–1.00
Manganese	Mn	0.67–0.90
Nickel	Ni	≤0.10
Iron	Fe	≤0.75
Carbon	C	≤0.35
Nitrogen	N	≤0.25

**Table 2:** Process parameters of LPBF.

*P* (W)	$P_{d}$ (μm)	$E_{t}$ (μs)	*V* (m/s)	*E*_v_ (J/mm^3^)
175	100	110	0.91	36
		80	1.25	22
		50	2.00	10
	70	110	0.64	52
		80	0.88	31
		50	1.40	14
	40	110	0.36	90
		80	0.50	55
		50	0.80	25
200	100	110	0.91	41
		80	1.25	25
		50	2.00	11
	70	110	0.64	59
		80	0.88	36
		50	1.40	16
	40	110	0.36	103
		80	0.50	63
		50	0.80	28
250	100	110	0.91	52
		80	1.25	31
		50	2.00	14
	70	110	0.64	74
		80	0.88	45
		50	1.40	20
	40	110	0.36	129
		80	0.50	78
		50	0.80	35

## Data Availability

Data are contained within the article.
